# Development and evaluation of bidirectional LSTM freeway traffic forecasting models using simulation data

**DOI:** 10.1038/s41598-021-03282-z

**Published:** 2021-12-13

**Authors:** Rusul L. Abduljabbar, Hussein Dia, Pei-Wei Tsai

**Affiliations:** 1 Department of Civil and Construction Engineering, Swinburne University of Technology, Melbourne, Australia; 2 Department of Computer Science and Software Engineering, Swinburne University of Technology, Melbourne, Australia

**Keywords:** Civil engineering, Sustainability, Information technology

## Abstract

Long short-term memory (LSTM) models provide high predictive performance through their ability to recognize longer sequences of time series data. More recently, bidirectional deep learning models (BiLSTM) have extended the LSTM capabilities by training the input data twice in forward and backward directions. In this paper, BiLSTM short term traffic forecasting models have been developed and evaluated using data from a calibrated micro-simulation model for a congested freeway in Melbourne, Australia. The simulation model was extensively calibrated and validated to a high degree of accuracy using field data collected from 55 detectors on the freeway. The base year simulation model was then used to generate loop detector data including speed, flow and occupancy which were used to develop and compare a number of LSTM models for short-term traffic prediction up to 60 min into the future. The modelling results showed that BiLSTM outperformed other predictive models for multiple prediction horizons for base year conditions. The simulation model was then adapted for future year scenarios where the traffic demand was increased by 25–100 percent to reflect potential future increases in traffic demands. The results showed superior performance of BiLSTM for multiple prediction horizons for all traffic variables.

## Introduction

The research on short-term traffic prediction models have been increased extensively in recent years to improve transport management^1^. An accurate prediction model can play an important role in optimizing freeway operations and avoiding traffic breakdowns. These models have been developed using simulated data or historical field data extracted from detectors attached along the roads. Then, these data become an input to statistical techniques and Artificial Intelligence (AI) based on machine learning models for a short-term traffic predictions^2,3^. However, the rapid development of big data and complex computational intelligence has created AI models (i.e. deep learning models) that can capture future traffic patterns more accurately than statistical models. An example of recent models are the Uni-directional long short term memory (Uni-LSTM) recurrent neural network and its extension Bidirectional long short term memory (BiLSTM). Previous research has shown that Uni-LSTM models are effective in handling long-term dependencies as they remember useful information from inputs that have already passed through using “additional gates” incorporated in their architectures^4–6^. However, bidirectional LSTM (BiLSTM) models have been tested in more recent year which offer additional training capabilities with the output layer receiving information from past (backwards) and future (forward) instances simultaneously providing better prediction accuracy^7–10^. In this paper, we assess the performance of BiLSTM for different time horizons using simulated data of count (flow), speed and occupancy (percentage of time vehicles occupy the loop detectors space which is a surrogate measure for density) from a calibrated and validated simulation model for the Eastern Freeway in Melbourne, Australia. The model was extensively calibrated and validated using field data collected from 55 sensors (indictive loop detectors) located along the freeway’s mainline from July 1, 2016 to August 31, 2016. This paper aims to demonstrate the feasibility of using advanced AI-techniques based on Deep Learning BiLSTM architectures to predict traffic count, speed and occupancy for multiple prediction horizons. The paper also provides a comparative performance evaluation of both Uni-LSTM and BiLSTM models based on the same set of simulated data and investigates whether BiLSTM models achieve good prediction accuracies for different traffic variables for multiple prediction horizons. The paper also validates the performance of developed models on future traffic scenarios when the traffic demand increases by 25%, 50%, 75% and 100% which makes this work a valuable contribution to knowledge in the Intelligent Transport Systems and network operations fields. Hence, it would provide road operators and transport agencies with confidence that this model can be adapted to future traffic patterns. Importantly, to the best of our knowledge, there has been limited research targeting the application of BiLSTM models for traffic prediction for multiple short-term prediction horizons, and this paper serves as a reference point to demonstrate their robust performance compared to Uni-LSTM models.

This paper is organised as follows: “Literature review” section provides a scan of previous research work. “Methodology” section presents the methodology including model calibration, data collection, modelling frameworks and modelling results. “Summary of results” section presents the conclusions and future research directions.

## Literature review

Short-term Traffic prediction plays an important role in the success of Intelligent Transport Systems (ITS) particularly for travel information systems, adaptive traffic management systems, public transportation scheduling and commercial vehicle operations^1,2,11^. Methodologies used in traffic prediction research can be divided into parametric and non-parametric approaches. The first approach include examples of linear models such as Autoregressive Integrated Moving Average Model (ARIMA), seasonal ARIMA, i.e. SARIMA model, exponential smoothing model, and ARIMA with Kalman Filter (KF)^12–16^. These models fail to capture the dynamic traffic patterns when compared to non-parametric methods. Non-parametric methods can handle the stochastic pattern and the noise in traffic input data for example deep learning neural network models which have been used to predict future traffic speeds, travel times, and traffic flows in many research papers^4,6,17^. With the development of machine learning and deep learning technology, the related non-parametric models are widely used in prediction problems in recent research and applications such as cyber security^18^, Heterogeneous Traffic and Anomaly Detection^19^, QoS of Web service^20^, electric vehicles^21^, Blockchain-Based System^22^ and , real-time processing systems in maritime sector^23^. The accuracy of these models is better than parametric models. For the purposes of this paper, we conducted a literature scan focused on short term traffic prediction using deep learning BiLSTM models which have been recently reported in the traffic data prediction field. The following search criterial was used in Scopus under “titles, keywords or abstracts”:

(("traffic prediction" OR "traffic forecast*" OR "transport prediction" OR "transport forecast*" OR "traffic speed prediction" OR "traffic Speed forecast*" OR "traffic flow prediction" OR "traffic flow forecast*" OR “travel time prediction" OR "travel time forecast*") AND ("BILSTM" OR "BI-LSTM" OR "Bidirectional LSTM" OR "Bi-Directional LSTM" OR "Bidirectional Long Short Term Memory")).

This search criterion resulted in 28 documents including 15 journal articles, 11 conference papers and 2 conference review papers. The authors have screened all the documents and excluded 5 that were more related to mobile computing instead of traffic prediction. Hence, only 23 documents were analysed for this literature review. The results showed that six papers were published in 2021; 12 papers were published in 2020; three papers were published in 2019, and one paper was published in 2018 and 2017, respectively. In terms of the most influential publications based on citations records, Table 1 represents the top 8 papers that use BiLSTM model in traffic prediction (excluding self-citations of all authors).

**Table 1:** Top cited documents using bilstm models.

Document Title	Publication Year	Authors	Journal Title	Citations per year				Total citations
				2018	2019	2020	2021	
Short-term traffic flow prediction with Conv-LSTM	2017	Liu Y., Zheng H., Feng X., Chen Z	2017 9th International Conference on Wireless Communications and Signal Processing, WCSP 2017—Proceedings	4	15	35	10	64
Traffic speed prediction for urban transportation network: A path based deep learning approach	2019	Wang J., Chen R., He Z	Transportation Research Part C: Emerging Technologies	1	10	22	9	42
Stacked bidirectional and unidirectional LSTM recurrent neural network for forecasting network-wide traffic state with missing values	2020	Cui Z., Ke R., Pu Z., Wang Y	Transportation Research Part C: Emerging Technologies	0	0	5	3	8
Short-term Traffic Flow Prediction Based on PCC-BiLSTM	2020	Zou H., Wu Y., Zhang H., Zhan Y	Proceedings—2020 International Conference on Computer Engineering and Application, ICCEA 2020	0	0	1	1	2
Urban traffic flow online prediction based on multi-component attention mechanism	2020	Sun B., Sun T., Zhang Y., Jiao P	IET Intelligent Transport Systems	0	0	0	1	1
Bidirectional Spatial–Temporal Network for Traffic Prediction with Multisource Data	2020	Sun T., Yang C., Han K., Ma W., Zhang F	Transportation Research Record	0	0	0	1	1
DeepBSTN: A Deep Bidirection Network Model for Urban Traffic Prediction	2019	Lu M., Pang J., Li J	Proceedings—5th International Conference on Big Data Computing and Communications, BIGCOM 2019	0	0	0	1	1
P-DBL: A deep traffic flow prediction architecture based on trajectory data	2018	Wang J., Xu X., He J., Li L	Lecture Notes in Computer Science	0	0	1	0	1

The most cited paper was a conference paper published by Liu^24^. The authors used BiLSTM model to extract periodic features of traffic flow to improve the spatial and temporal traffic flow prediction from Convolutional-LSTM model. The results showed that their proposed model provided better accuracies when compared to other models. However^25^, compared Convolutional-LSTM models against BiLSTM models and showed that they provided better accuracy for traffic flow prediction. The second paper examined paths in road network for traffic speed forecasting using BiLSTM^26^. The model exploited the spatial–temporal feature along each selected path and achieved better prediction performance when compared with other models. Similarly^27^, focused on A Path-based Speed Prediction Neural Network to achieve speed predictions for a given path and attributes to provide large-scale optimised paths speed information for both transport authorities and travellers. The third paper used BiLSTM models for a network-wide traffic state prediction and added imputation units in the model to fill the missing values in the spatial–temporal input data with the results demonstrating an improvement in prediction accuracy^28^. Moreover, Bi-LSTM models were used to extract temporal features of traffic flow and were combined with spatial features to improve short-term flow prediction^9^. The importance of BiLSTM models in capturing complex non-linear urban traffic flow features was also investigated by^10^ which showed improved predictions. Similar work which focused on traffic flow prediction using this model was conducted by^29–31^. Reference^32^ used multisource data of speed and weather for future spatial and temporal speed prediction. Similarly^33,34^, used precipitation information to provide accurate traffic flow prediction using BiLSTM model. Reference^35^ captured complex spatial–temporal correlation by using BiLSTM model for traffic flow prediction. Likewise, traffic flow-related environmental factors were taken into consideration to improve the accuracy of traffic flow prediction using BiLSTM models^36^. Other research also demonstrated an improved traffic flow prediction accuracy when using this model under connected and automated vehicle environments^37,38^.

LSTM and BiLSTM models were previously mentioned in other publications and used to forecast future traffic speeds^39^, traffic flows^6^ and travel times^40^. For example, an LSTM model was developed to predict future speeds with better prediction accuracy when compared to classical methods^39^. In another study, the authors showed the superior performance of LSTM models for irregular travel time prediction models as the error for 1-step-ahead prediction was relatively small^40^. Another study showed the capability of LSTM model in flow prediction when compared to other models for multiple prediction horizons in the future^6^. In addition, LSTM models have been developed for car-following models to predict acceleration and deceleration on different road hierarchies^41^. LSTM model has also been investigated where the dependency relationships of time series data were fully considered, and the results showed a very good performance with a small prediction error when compared with other models^42^. Other authors developed an end to-end deep learning with 1 BiLSTM layer for future traffic flow prediction, and the results showed that the model was capable of solving stochastic flow characteristics and overcoming overfitting problems^43^. Similarly, multiple layers of BiLSTM and LSTM models were investigated to predict network wide traffic speeds resulting in superior performance compared to other models^44^. In another study, multiple BiLSTM models were developed providing good accuracies for urban traffic prediction^45^. Other authors have also used LSTM and RNN approaches for speed prediction models under various urban driving conditions with accurate results^46^. LSTM and gated recurrent units (GRUs) models were also investigated in a recent study to predict the general condition of driving speed in consideration of the road geometry and temporal evolution of traffic demand. The results showed superior LSTM model performance compared to regression models^47^. Correspondingly, superior model performance has been shown from using LSTM and GRU models when compared to ARIMA and support vector regression (SVR) models for the track flow prediction^48^. Furthermore, a variational long short-term memory encoder was tested for future traffic flow prediction with good results in comparison to other conventional methods^49^. In a similar study, a long short-term memorygenetic algorithm support vector regression (LSTMGASVR) algorithm was investigated to predict future traffic flows with a superior performance in comparison to other models^50^. Other authors have also tested LSTM models for continuous traffic informational collection and proved its ability to provide accurate information of flow^51^. Also, LSTM models have been developed in another study on traffic flow short-term prediction and the results showed high prediction accuracies for flow data^52^. Similarly, other authors have documented a superior performance when combining ARIMA and long short-term memory (LSTM) neural networks for short-term traffic flow prediction^53^. Finally, a type-2 fuzzy LSTM (T2F-LSTM) model was developed for long-term prediction and extraction of spatial–temporal characteristics of traffic volumes and showed high prediction accuracies in comparison to other models^54^. It can be noticed that there is more focus in these studies on exploring the spatial and temporal traffic features when predicting traffic conditions using the BiLSTMmodel^55–59^. However, few studies have explored the feasibility of this type of model to be validated or transferred (without retraining) to an independent dataset from a different freeway^60^ or in the case of this paper, validate the model against future traffic scenarios where the demand is expected to increase to up to 100% in the future. Also, this paper tests the model on multiple prediction horizons on multiple traffic variables such as speed, flow and occupancy using data generated from a calibrated freeway model which hasn’t been established in any previous literature on the topic.

## Methodology

This work relied on generating simulated data for model development and evaluation. The freeway under consideration was simulated using the Aimsun traffic simulation software^61^. Substantial effort was devoted for model calibration and validation to ensure that the simulation model outputs replicated real-world behaviour. The main advantage for using simulation models is the ability to generate large amount of data that represent different traffic conditions including incidents, shockwaves and other edge-case behaviours that are difficult to capture or replicate in the field. For brevity, we only present model calibration and validation and refer the readers to other references for more details about the theoretical aspects of traffic simulation and steps for model development^62–64^.

### Model calibration

**Figure 1:**
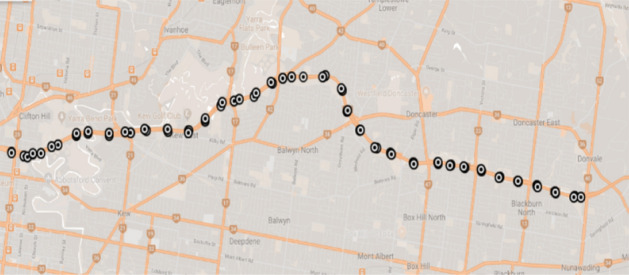
Detections location on the mainstream of Eastern Freeway.

The real-life data used for calibration was collected from inductive loops embedded along the Eastern Freeway in Melbourne/Australia (Fig. 1). The data was collected for a period of two months from 1/07/2016 to 30/08/2016 for both the eastbound and westbound directions. The data covered all 24 h of each day and was aggregated at 1-min intervals across all lanes at each site. Due to detector fault or unreliable results, some detector data was not used in the calibration process. In total, 55 detectors were used for the calibration including 26 detectors for the eastbound and 29 detectors for the westbound directions. The models was then calibrated for the peak hour period 6:00–9:00 AM.

A large number of parameters play an important role in model calibration and need to be specified accurately. These include basis vehicle-specific parameters such as length, width and maximum desired speed^61^. As well as more complex and dynamic model parameters such as speed acceptance (degree of driver’s compliance with speed limits), clearance (distance from the vehicle to the vehicle ahead) and maximum give way time (threshold in seconds beyond which a driver can no longer wait for a gap in traffic to perform a certain manoeuvre like a lane change). In the microscopic model parameters, maximum acceleration, maximum deceleration and sensitivity factors are also considered.

Modelling the dynamic behaviour is essential in the calibration process. Vehicle dynamic behaviour is presented by the type of vehicle, vehicle’s size, maximum acceleration/deceleration and driver behaviour. These parameters directly impact traffic flow in the network. Other factors such as headway, response time, gap acceptance threshold for lane changing, or distance for lane changing also impacts the flow of vehicles in the simulated network. Once these parameters are specified, the mode’s calibration can be evaluated using a number of pre-determined measures that include GEH and RMSE key performance indicators. For a detailed coverage of the processes and requirements for model calibration, the reader is referred to^65,66^.

1. GEH
  The **GEH** is a measure used to quantify traffic volume differences between observed and simulated data. It is named after the inventor Geoffrey E. Havers^67–69^. The GEH statistic is defined as:
  1 $$ GEH = \sqrt {\frac{{2(m - o)^{2} }}{{\left( {m + o} \right)}}} $$
  where m is the modelled hourly count; and o the observed hourly count
  In Aimsun, the GEH discrete statistic classifies the GEH values in a number of categories, which include:
  GEH < 5: Good fit.
  GEH 5 – 10: Requires further investigation.
  GEH > 10: Poor fit: Unacceptable.
2. ROOT MEAN SQUARE ERROR “RMSE”
  RMSE is a standard measure that estimates the error of predictions for detector *i* using the following equation^64,65^:
  2 $$ RMS_{i} = \sqrt {\frac{1}{m}\mathop \sum \limits_{j = 1}^{m} \left( {s_{{ij - P_{ij} }} } \right)^{2} } $$
  where, $s_{ij}$ is the actual measurement for detector *i* at sampling interval *j.*
  $p_{ij}$ is the simulated measurement for detector $i$ at sampling interval $j$.

### Calibration results

**Figure 2:**
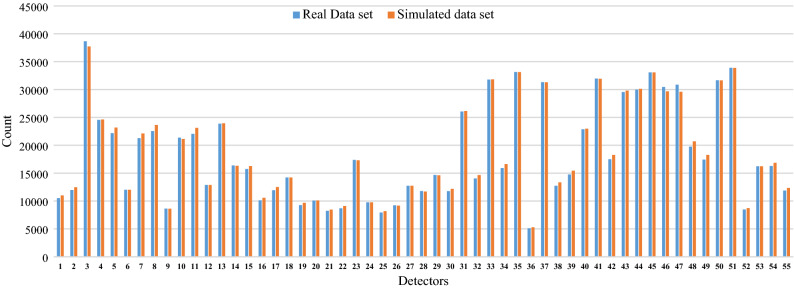
Traffic flow calibration results.

**Figure 3:**
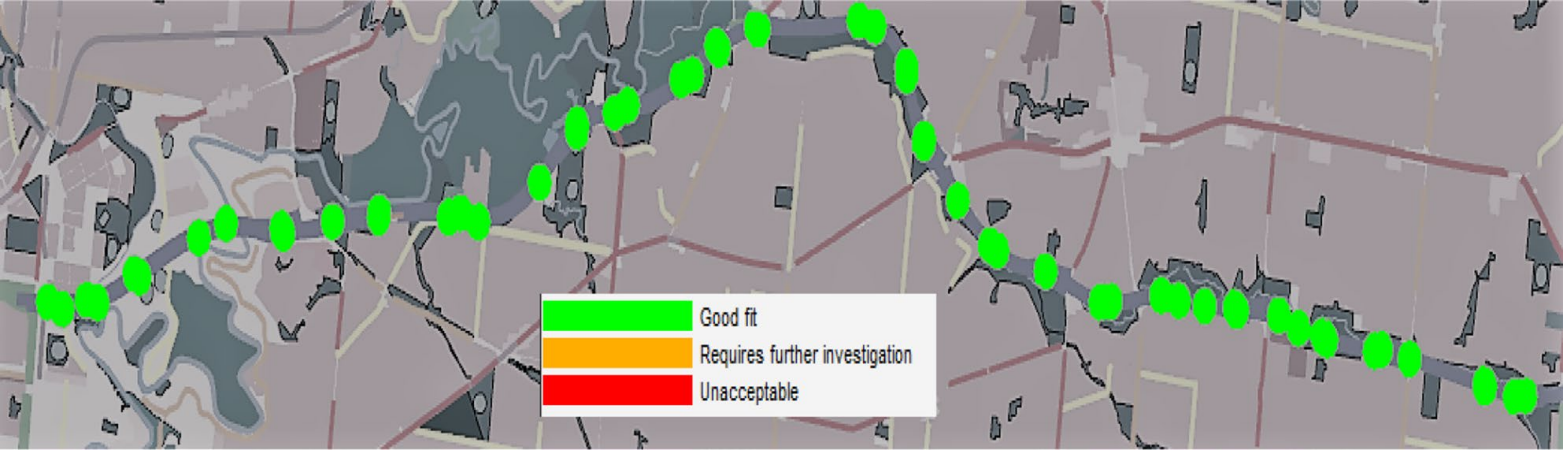
The Discrete GEH Statistic view mode for count.

Figure 2 presents the calibration results and shows a comparison between the real-world field data and the simulated data generated from Aimsun. The blue bar shows the real vehicle count values collected from the field while the orange bar represents the simulated count values collected from the AIMSUN model. The two bars share very close count values demonstrating a good model calibration against real-life datasets. Figure 3 shows the base view mode GEH Statistic values represented in circles for each detector. A red circle means that GEH value is unacceptable, an amber circle means that GEH values needs further investigation and a green circle means that the model is a good fit. The results show that the GEH values of all detectors are shown as a green circles indicating that all detectors have a value of less than 5 demonstrating a good fit model.

**Figure 4:**
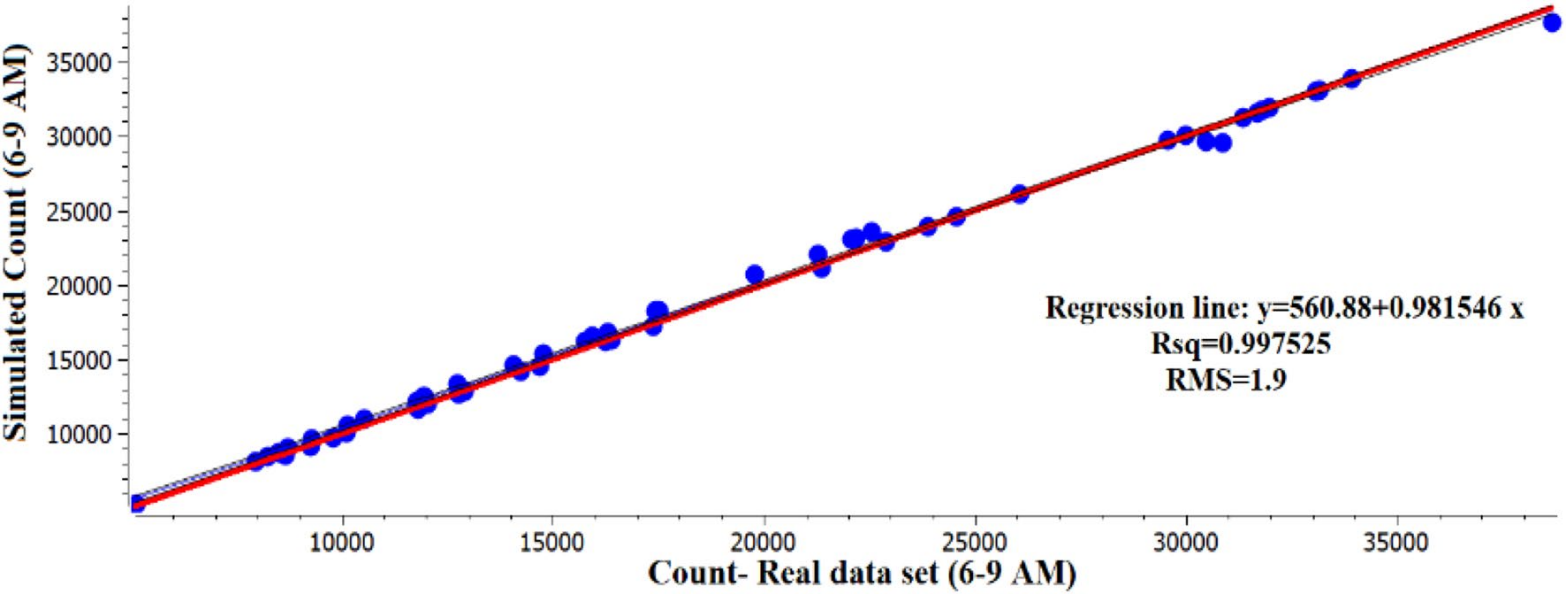
Regression analysis of the real data set for count vs simulated count.

Finally, Fig. 4 shows a regression of the real-world data versus simulated data which also demonstrates high model performance as evidenced by the high coefficient of determination (R-square) and the low RMS error. This provides confidence that the model has been calibrated to a reasonable degree of accuracy in terms of its ability to replicate real-world conditions and that it can be used with high levels of certainty in this research to generate data that can be used for development of prediction models.

### BiLSTM model developement

This section of the paper presents the study methodology including data collection, model development, evaluation tests and analyses.

#### Data for model development

**Figure 5:**
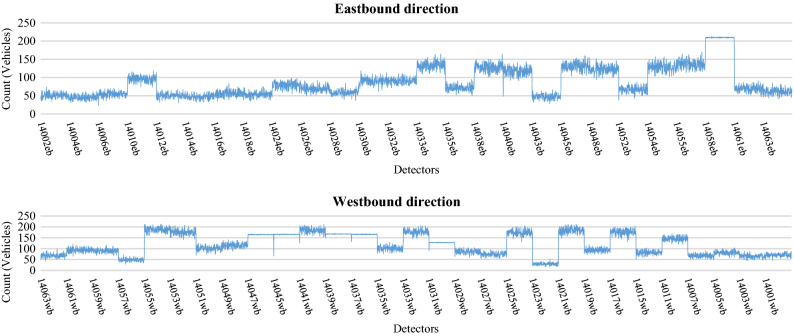
Simulated count data used for the eastbound and westbound directions.

**Figure 6:**
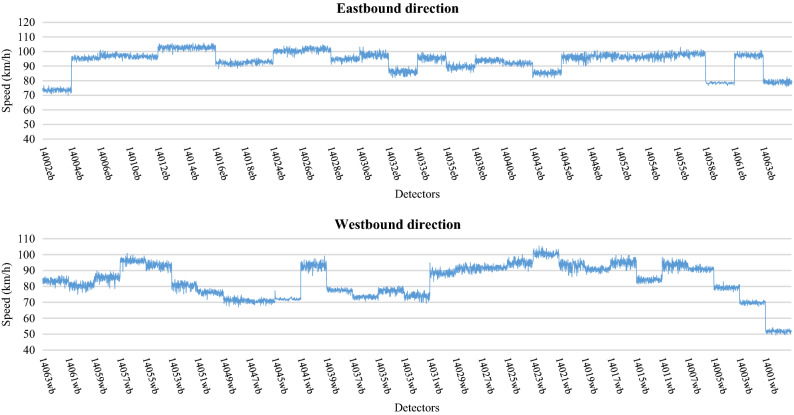
Simulated speed data used for eastbound and westbound directions.

**Figure 7:**
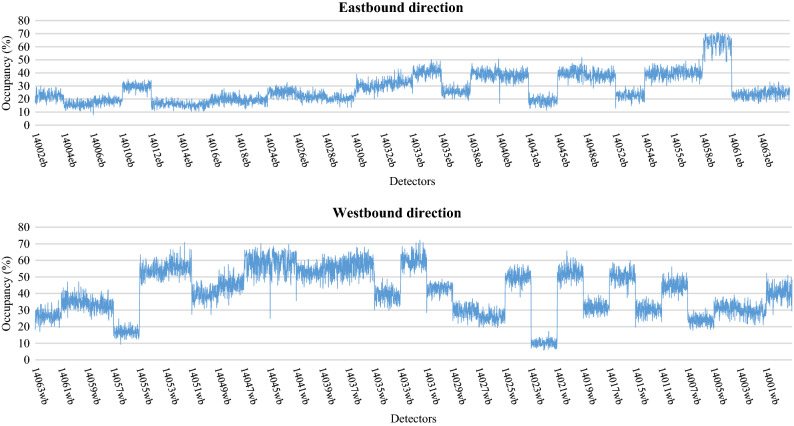
Simulated Occupancy data used for eastbound and westbound directions.

Neural network applications require large amounts of data for model development^61,67^. The data is typically divided into a training data set used for model calibration, and a testing data set used for model verification. The training data usually comprises the largest set of observations and is used to train the model to perform a desired action. Using this data, a neural network application learns the patterns of association between inputs and outputs, and forms a relationship between the different variables. The validity of the model is tested on an independent data set not used in model training, referred to as the testing data set. The real-life data was collected from inductive loops embedded along the Eastern Freeway in Melbourne, Australia. These data were used for the calibration and validation process of the simulation model. The model was successfully calibrated to a high degree of accuracy representing the baseline scenario situation for Eastern Freeway. After that, multiple data were generated from the baseline calibrated scenario and then used for model development. These data included traffic volumes, speed and occupancy measurements (percent of time a vehicle spends on top of the loop detectors) collected during peak hours from (6–9) AM. These data were generated from the baseline scenario model at 1-min intervals with a total of 9,900 observations collected for each traffic feature. Figures 5, 6 and 7 show typical patterns of traffic counts, speeds and occupancy data respectively, for each detector station.

#### Modelling framework

**Figure 8:**
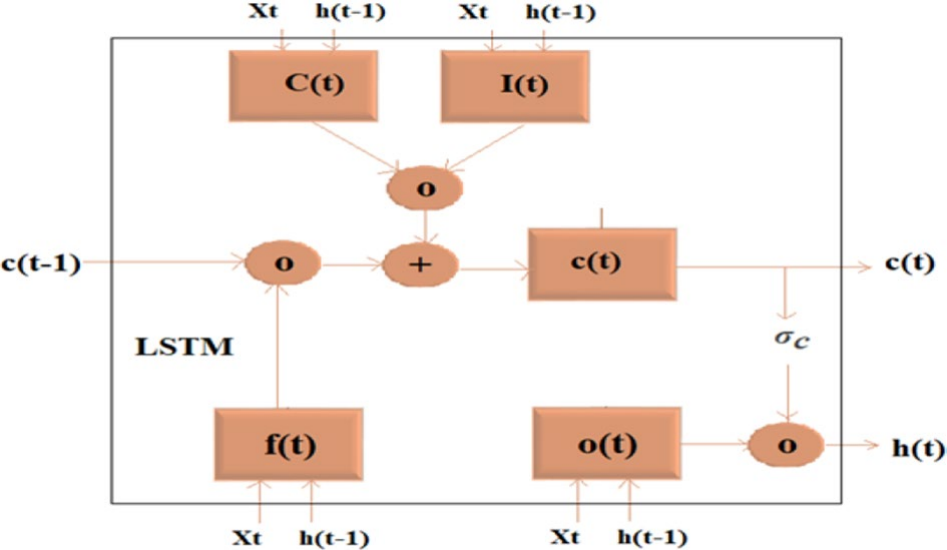
LSTM Architecture^60^.

**Figure 9:**
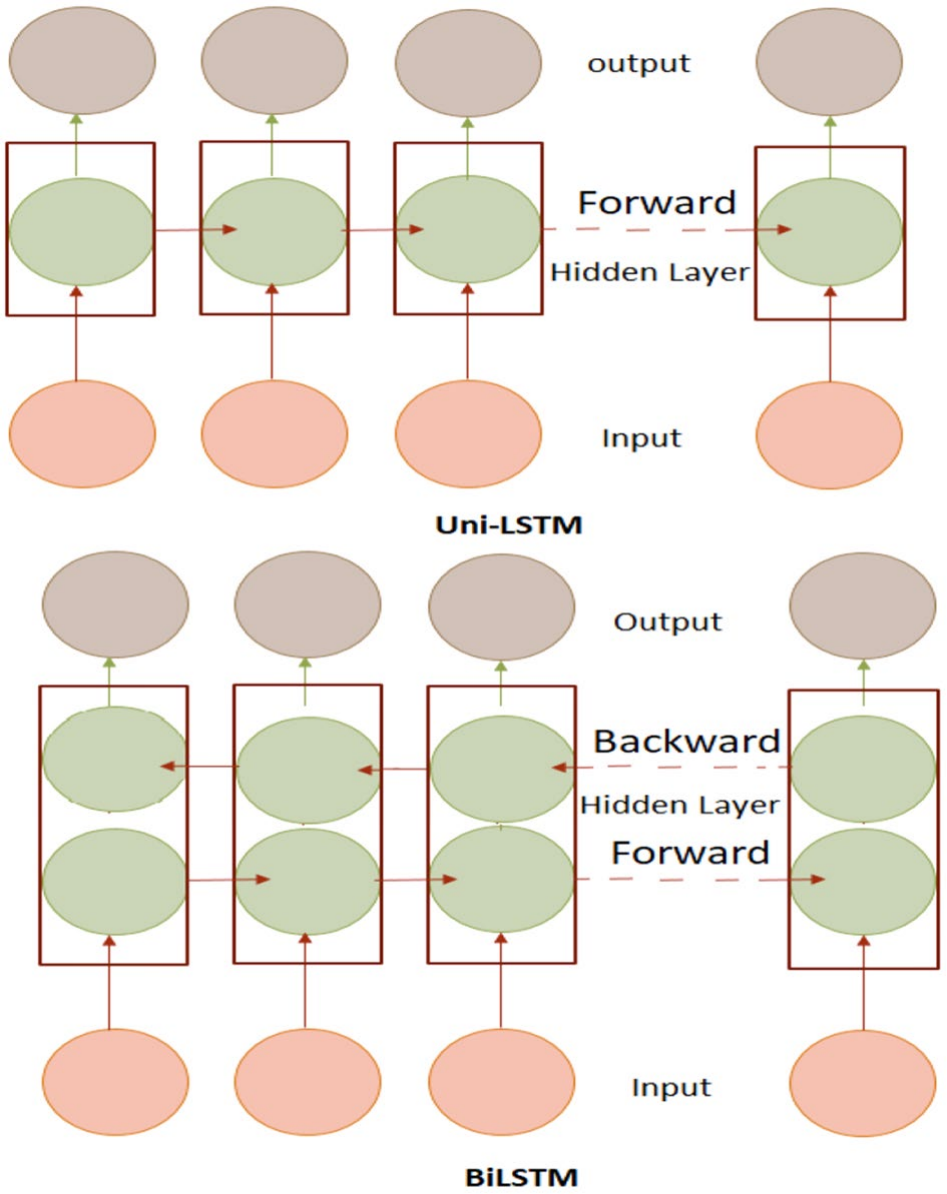
Uni-LSTM/ BiLSTM Architecture^60^.

Unidirectional LSTM received considerable attention in recent years for its superior performance compared to the state-of-art Recurrent Neural Networks (RNNs). Even though RNNs provide good accuracy, they have been found to underperform for long-term memory as RNNs are unable to use information from the distant past. Also, LSTM can learn patterns with long dependencies when compared with traditional RNNs^70^. The inclusion of additional training has resulted in some model extensions of LSTM known as Bidirectional LSTM (BiLSTM). This model trains the input time series data twice through forward and backward directions as shown in in Figs. 8 and 9**.**

In these models, the following formulae are used to calculate the predicted values^60,71^:

3 $$ {\text{Input gate(I}}_{{\text{t}}} ) = {\upsigma }_{{\text{g}}} \left( {{\text{W}}_{{\text{i}}} {\text{X}}_{{\text{t}}} + {\text{R}}_{{\text{i}}} {\text{h}}_{{{\text{t}} - 1}} + {\text{b}}_{{\text{i}}} } \right). $$

4 $$ {\text{Forget gate}}\;\left( {{\text{f}}_{{\text{t}}} } \right) = {\upsigma }_{{\text{g}}} \left( {{\text{W}}_{{\text{f}}} {\text{X}}_{{\text{t}}} + {\text{R}}_{{\text{f}}} {\text{h}}_{{{\text{t}} - 1}} + {\text{b}}_{{\text{f}}} } \right). $$

5 $$ {\text{Cell state}}\;{\text{(C}}_{{\text{t}}} ) = {\upsigma }_{{\text{c}}} \left( {{\text{W}}_{{\text{c}}} {\text{X}}_{{\text{t}}} + {\text{R}}_{{\text{c}}} {\text{h}}_{{{\text{t}} - 1}} + {\text{b}}_{{\text{c}}} } \right). $$

6 $$ {\text{Output gate}}\;{\text{(o}}_{{\text{t}}} ) = {\upsigma }_{{\text{g}}} \left( {{\text{W}}_{{\text{o}}} {\text{X}}_{{\text{t}}} + {\text{R}}_{{\text{o}}} {\text{h}}_{{{\text{t}} - 1}} + {\text{b}}_{{\text{o}}} } \right). $$

where σg is the gate activation function, $W_{i} ,W_{f} ,W_{c} { }\,\,and\,\,{ }W_{o}$. are input weight matrices.

$$R_{i} ,{ }R_{f} ,R_{c} { }\,\,and\,\,{ }R_{o}$$. Are recurrent weight matrices, $$X_{t}$$. is the input $$h_{t - 1} $$ put at the previous time (t − 1). $$b_{i} ,b_{f} ,b_{c} { }\,\,and\,\,{ }b_{{o{ }}}$$ Are bias vectors. The “input gate” specifies new input to the cell state, the “forget gate” determines how much of the prior memory values should be removed from the “cell state”^60,71^ and the “cell state” and “output gate” of the LSTM at time t is calculated as follows:

7 $$ {\text{C}} = {\text{ ft}} \odot {\text{ct}} - {1} + {\text{it}} \odot {\text{gt}} $$

8 $$ {\text{Ht }} = {\text{ ot}} \odot \sigma {\text{c}}\left( {{\text{ct}}} \right) $$

where ⊙ denotes the Hadamard product (element-wise multiplication of vectors).

In this work, the Unidirectional and Bidirectional LSTM networks were implemented in Matlab R2020b. Similar to^60^, first the data was arranged in two columns: the first column corresponds to speed/flow at time (t) and the second column corresponds to the expected output (t + n) where n ranges from 5 to 60 min into the future. Then, the data were partitioned into training and testing sets. The models were trained on the first 60% of the sequence and tested on the last 40%. To prevent model overfitting, the training/testing data were standardised to have zero mean and unit variance^60^. The LSTM networks were created using four layers: Sequence Input Layer (number of Features = 1), Uni-LSTM/ BiLSTM Layers (number of Hidden Units = 300), fully Connected Layer (number of Responses = 1) and a Regression Layer. The model hyper parameter settings are presented in Table 2. The same parameters were optimised by the authors for^60^ and they achieved high prediction accuracies. The tanh and sigmoid functions were used for state and gate activation functions, respectively. The LSTM experiments were also implemented in Matlab R2020b with the Deep Learning Toolbox functions of trainNetwork, training Options, and predictAndUpdateState.

**Table 2:** Model hyper parameters for UNI-LSTM and BiLSTM^60^.

Parameters	Settings
Gradient Decay Factor	0.9
Initial Learning Rate	0.005
Minimum Batch Size	128
Maximum Epochs	300
Training Optimizer	Adaptive Moment Estimation Optimizer
Dropping Learning Rate During Training	Piecewise
Learning Rate Drop Period	125
Factor for Learning Rate Dropping	0.2

To evaluate BiLSTM prediction robustness, multiple machine learning systems were evaluated using the same data set. These included: Uni-LSTM, Recurrent Neural Networks (RNNs), ELMAN, Deep Learning Backpropagation (DLBP) neural networks.

These models have been widely used for future traffic forecasts, as shown in the example papers provided in the literature review section above. The models reported in this paper were developed using NeuralWorks Professional and MATLAB. NeuralWorks Professional is an Artificial Neural Network commercial package and development system^67^. Uni-LSTM consisted of 4 Layers: Input layer, number of Hidden Units (300 units), fully Connected Layer (number of Responses = 1) and a Regression Layer. The model hyper parameter settings are similar to BiLSTM model which is presented in Table 2. The tanh and sigmoid functions were also used for state and gate activation functions, respectively for a fair comparison between the two models. RNNs and ELMAN are feedforward neural networks that perform well with time series forecasting data. The parameters used for this experiment were: hidden layers (1) with (5) neurons, activation function (tanh), learn rule (ext DBD) and epoch (770). The Backpropagation Neural Network is the most popular learning algorithm used to capture non-linear relationships and self-learning. The typical back-propagation network always has an input layer, an output layer and more than one hidden layer, which is referred to as “Deep Learning”. Each layer is fully connected to the succeeding layer. The implementation of the algorithm simply includes an input training pattern (feedforward), backpropagated error and weight adjustment. The parameters used for this experiment included 3 hidden layers with 4, 6, and 2 neurons. The transfer function is Tanh with a learning coefficient output = (0.15). The learning rule is Ext DBD with 100,000 iterations and a momentum of 0.4.

#### Model development results

In this section, BiLSTM is developed to predict future speed, traffic count and occupancy for up to 60 min into the future. As mentioned before, the simulated data from the calibrated freeway model were divided into 60% training data and 40% testing data. The BiLSTM model is evaluated against other models as shown in Table 3. The Mean Absolute Percentage Error (MAPE) is used to calculate the prediction accuracies for model comparison and evaluation for different time horizons. MAPE calculates the average absolute difference between the predicted output from the model (Y1) and expected true output (Y).

9 $$ {\text{MAPE }}\left( \% \right) \, = \left( {\frac{1}{n}\mathop \sum \limits_{i = 1}^{n} \frac{{\left| {Y - Y1} \right|}}{Y}} \right)*100 $$

10 $$ {\text{Accuracy }}\left( \% \right) \, = \, \left( {{1}00 \, {-}{\text{ MAPE}}} \right) $$

The count prediction results showed that BiLSTM achieve high prediction results up to 60 min into the future (Table 3). BiLSTM outperformed Uni-LSTM with accuracies above 93% up to 60 min. Accuracy improvements percentage of BiLSTM over Uni-LSTM were 5% for 5 min, 7% for 10 min, 9% for 15 min, 19% for 30 min, 25% and 35% for 45 and 60 min respectively. The improvement (%) is calculated as follows:

11 $$  {\text{Accuracy}}\;{\text{Improvement}}\;\left( \%  \right) = \frac{{{\text{Accuracy}}\;\left( {\text{\% }} \right)\;{\text{of}}\;{\text{BiLSTM}}\;{\text{model}} - {\text{Accuracy}}\;\left( {\text{\% }} \right)\;of\;UniLSTM\;model}}{{{\text{Accuracy}}\;\left( {\text{\% }} \right)\;of\;UniLSTM\;model}}  $$

Speed prediction results showed that BiLSTM achieved high prediction results up to 60 min into the future. BiLSTM outperformed Uni-LSTM with accuracies above 96% up to 60 min. Accuracy improvements percentage of BiLSTM over Uni-LSTM were small representing 1% for 5 min, 1% for 10 min, 1% for 15 min, 2% for 30 min, and 4% for 45-min prediction horizons. However, for 60-min prediction horizons, the accuracies from the two models were close (96.12% for BiLSTM and 95.98% for Uni-LSTM) shown in Table 4.

Similarly, occupancy prediction results showed that BiLSTM achieved high prediction results up to 60 min into the future. BiLSTM outperformed Uni-LSTM with accuracies above 92% up to 60 min. Accuracy improvement percentages of BiLSTM over Uni-LSTM were 9% for 5 min, 8% for 10 min, 7% for 15 min, 13% for 30 min, 11% and 15% for 45 and 60 min respectively as shown in Table 5.

**Table 3:** Count performance for different prediction horizons*.*

Prediction Horizons	Count (vehicles)					
	BP	ELMAN	RNN	UNI-LSTM	BiLSTM	Accuracy Improvement (%)
	Accuracy	Accuracy	Accuracy	Accuracy	Accuracy	BiLSTM over Uni-LSTM
5 Mins	88.43%	88.78%	87.21%	90.28%	95.21%	5.46%
10 Mins	87.11%	87.04%	85.51%	88.13%	94.45%	7.17%
15 Mins	84.18%	85.25%	84.09%	86.51%	93.94%	8.59%
30 Mins	80.09%	80.16%	79.04%	78.31%	93.32%	19.17%
45 Mins	75.70%	75.31%	74.58%	74.09%	92.95%	25.46%
60 Mins	69.47%	70.97%	69.95%	68.71%	93.04%	35.41%

**Table 4:** Speed performance for different prediction horizons.

Prediction Horizons	Speed (Km/h)					
	BP	ELMAN	RNN	UNI-LSTM	BiLSTM	Accuracy Improvement (%)
	Accuracy	Accuracy	Accuracy	Accuracy	Accuracy	BiLSTM over Uni-LSTM
5 Mins	95.91%	97.38%	95.84%	98.21%	98.88%	0.68%
10 Mins	96.49%	97.10%	93.24%	98.00%	98.82%	0.84%
15 Mins	96.45%	96.32%	94.85%	97.77%	98.65%	0.90%
30 Mins	96.35%	96.47%	94.26%	96.83%	98.41%	1.63%
45 Mins	95.68%	94.44%	90.51%	95.00%	98.46%	3.64%
60 Mins	93.61%	93.84%	92.02%	95.98%	96.12%	0.15%

**Table 5:** Occupancy performance for different prediction horizons.

Prediction Horizons	Occupancy (%)					
	BP	ELMAN	RNN	UNI-LSTM	BiLSTM	Accuracy Improvement (%)
	Accuracy	Accuracy	Accuracy	Accuracy	Accuracy	BiLSTM over Uni-LSTM
5 Mins	84.99%	87.71%	85.66%	89.96%	98.29%	9.26%
10 Mins	86.08%	86.49%	85.22%	88.22%	95.06%	7.75%
15 Mins	84.03%	85.26%	83.21%	87.71%	93.67%	6.80%
30 Mins	81.00%	81.02%	80.14%	82.18%	92.46%	12.51%
45 Mins	77.77%	78.01%	77.95%	82.76%	91.78%	10.90%
60 Mins	73.42%	73.93%	74.16%	79.50%	91.55%	15.16%

#### Future years traffic scenarios

**Figure 10:**
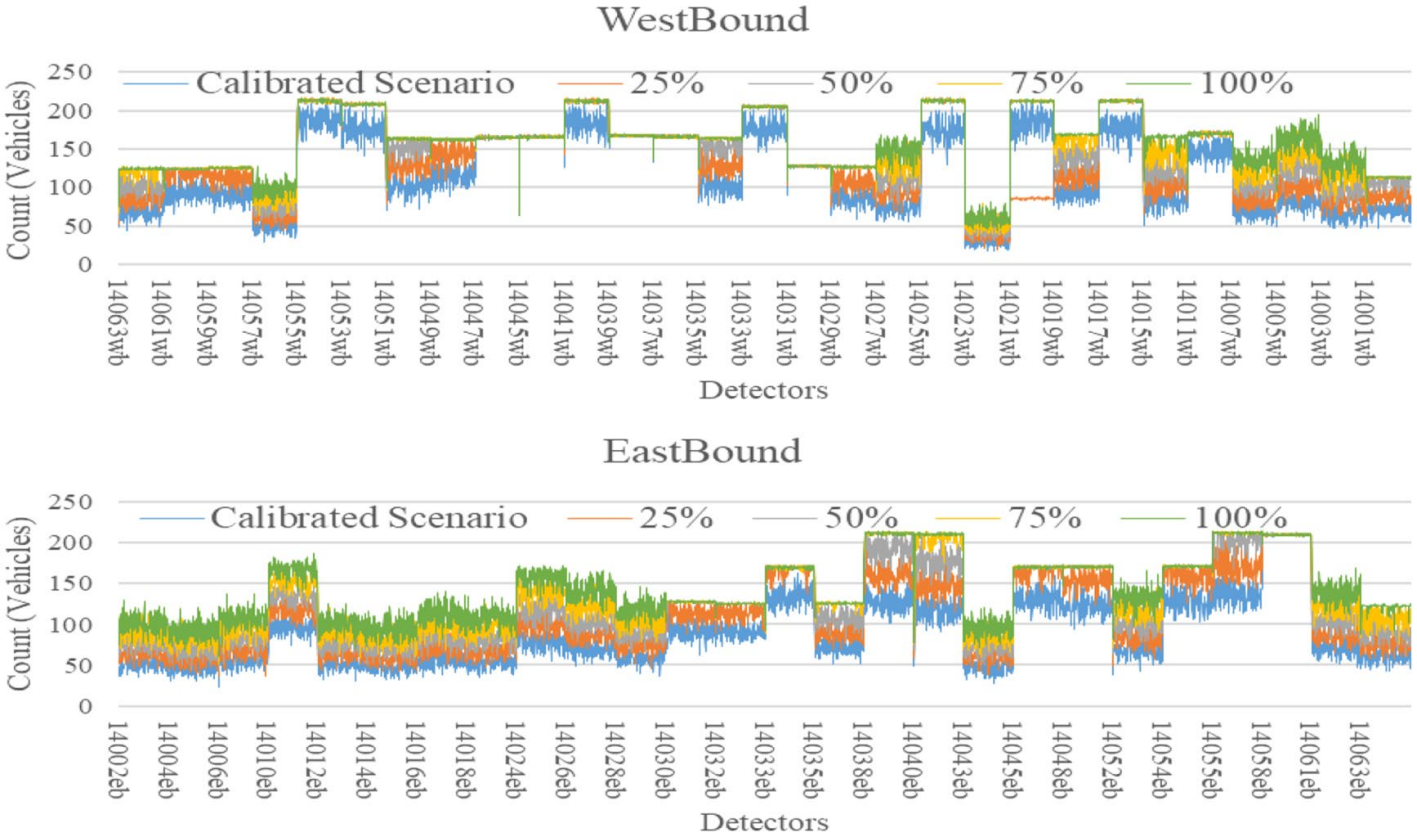
Future count data generated from Aimsun.

**Figure 11:**
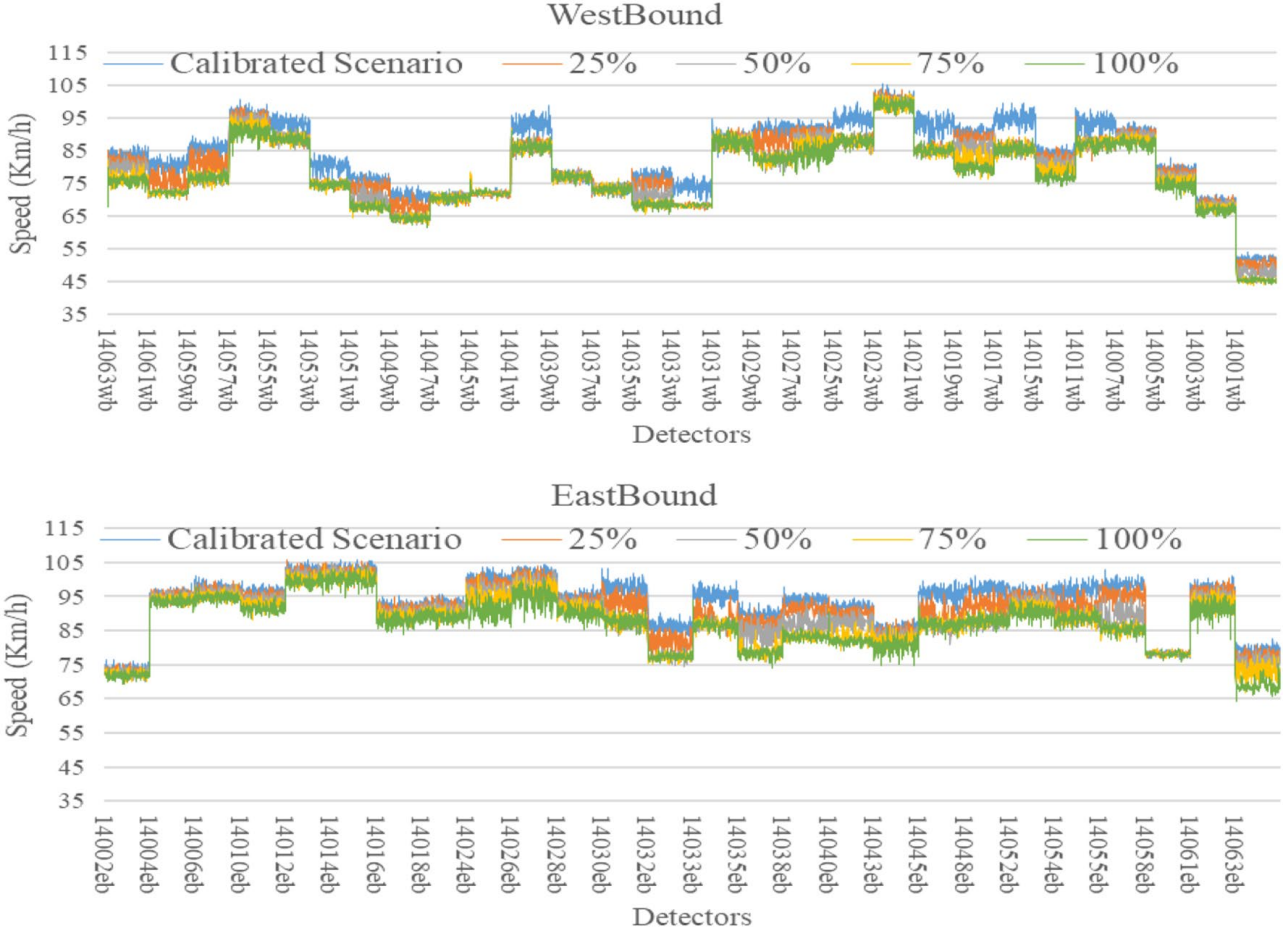
Future speed data generated from Aimsun.

**Figure 12:**
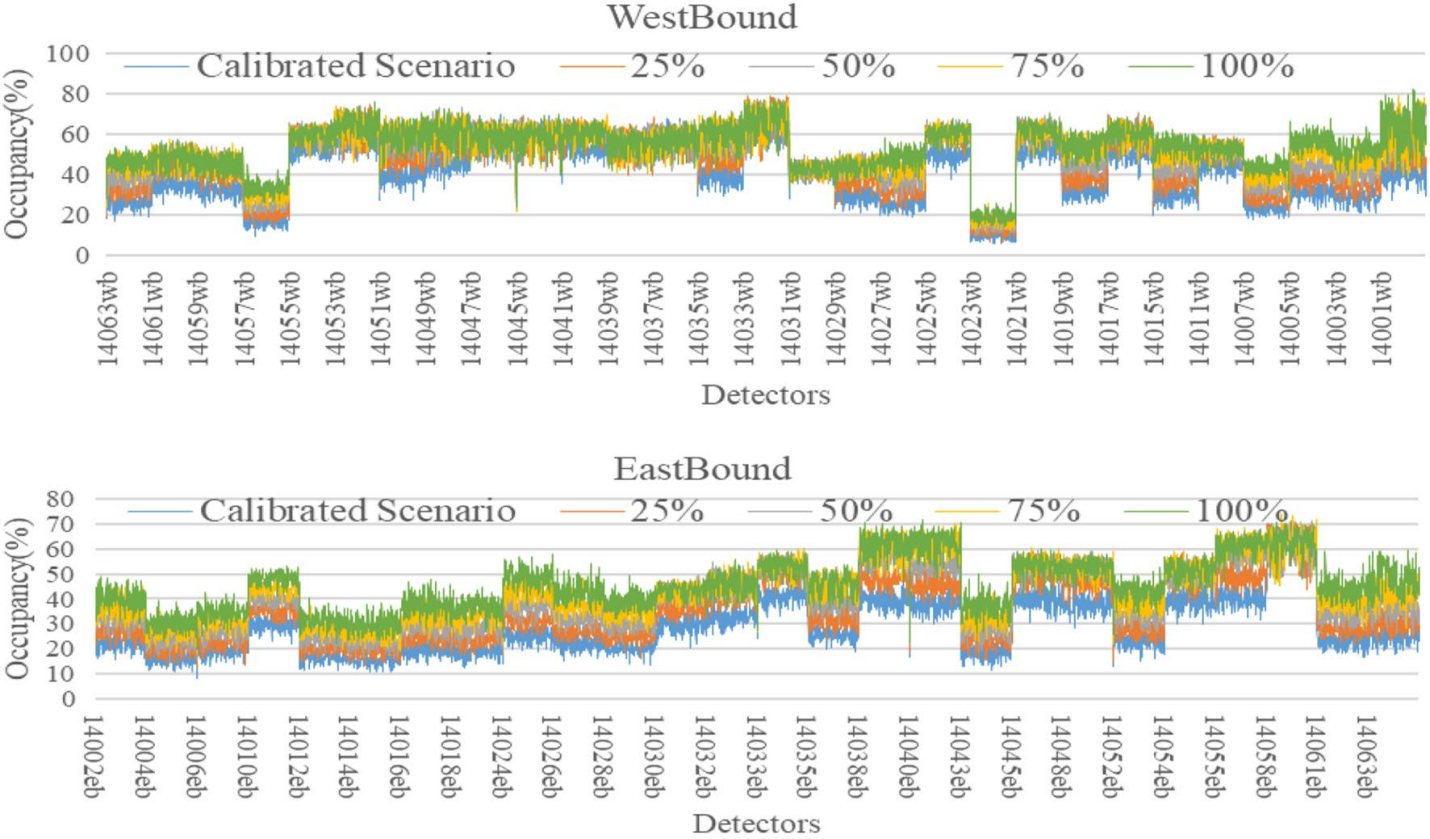
Future occupancy data generated from Aimsun.

The AIMSUN model used so far was calibrated for 2016 base year conditions and as shown before has proven its effectiveness as a short-term predictive model when compared with other models. The key advantage of simulation models is that they can be used to evaluate the impacts of traffic growth scenarios on road network performance. To demonstrate this, the traffic demand was increased by 25%, 50%, 75% and 100% to represent some future year traffic conditions. For each scenario, the same BiLSTM models were used (without re-training) for short-term forecasts up to 60 min into the future. A total of 9,900 observations were used for model development with 60% Training (5,940 observations) and 40% testing (3,960 observations). Then, observations were collected for each future traffic scenario and used for validation purposes without re-training the model. For example, Melbourne’s transport system handles 17 million trips per day and is expected to increase to 30 million per day by 2050^72^. Hence, it is important to develop a model that is able to cope with the future traffic demand changes. In the calibrated base scenario, the total number of vehicles passing through the freeway for three hours were 401,229 vehicles which is represented by the blue line in Figs. 10, 11 and 12 respectively. Then, the demand was assumed to be increased by 25% (orange line), 50% (grey line), 75% (yellow line) and 100% (green line). Therefore, the number of vehicles was increased to 501,536, 601,844, 702,151 and 802,458 vehicles respectively to reflect these future year increases. The data for all scenarios for traffic count, speed and occupancy are shown in the figures below for eastbound and westbound directions.

**Figure 13:**
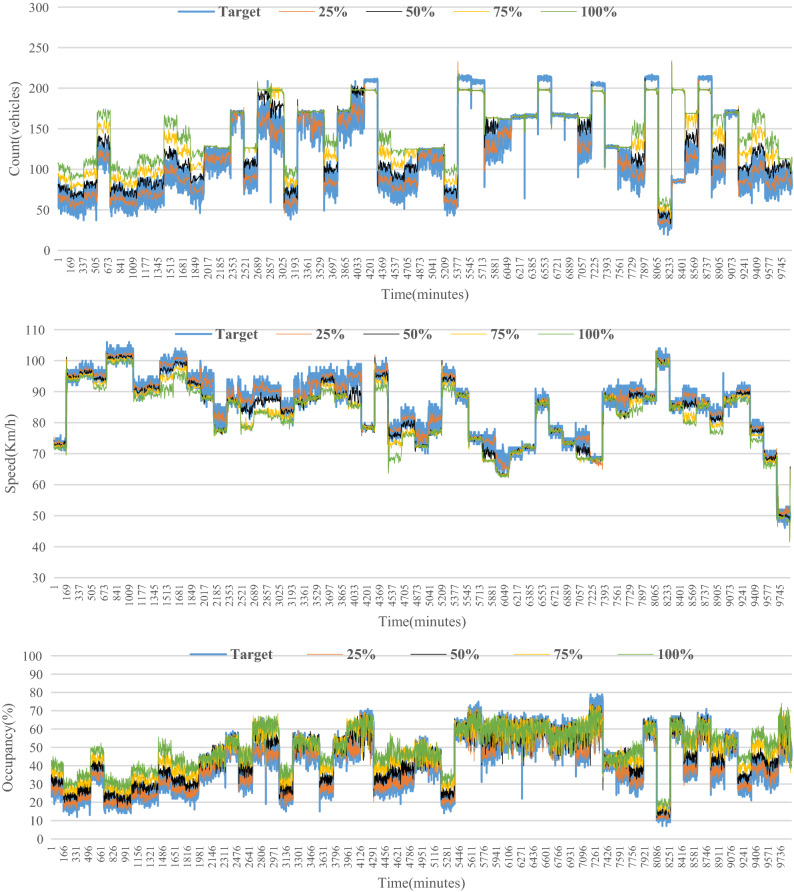
Count, speed and occupancy prediction results for all scenarios on 15 min prediction horizon.

#### Future traffic scenarios results

The results showed that BiLSTM is capable of an accurate prediction even for future traffic demands that are up to 100% more than base year demands. As can be seen in Table 6. When the model is validated without re-training, prediction accuracies for traffic volumes and speeds were above 90% for all future demand scenarios for prediction horizons up to 60 min into the future. For occupancy, the model was able to predict up to 45 min with an accuracy above 90% then performance decreased to 82–88 percent for 60-min prediction horizons. Figure 13 represents a prediction horizon of 15 min horizons in which the targeted data of traffic count, speed and occupancy were compared with predicted data generated from the BiLSTM model for all traffic demand scenarios. The blue line represents the targeted values for 15 min prediction horizons, the orange line represents a 25% increase in demand. whereas, 50%, 75% and 100% increase in demand were presented by the grey, yellow and green lines respectively. When the demand increases by 25%, the prediction accuracy for traffic counts between targeted and predicted values was 93%. The accuracy continues with high values of 95%, 95% and 96% when future traffic demand is increased by 50%, 75% and 100%. For speed, the 25% increase in demand resulted in 99% accuracy. When the demand increased by 50%, the model still achieved 99% accuracy. The error continues with same high accuracy of 99% when future traffic demand is increased by 75% and 100%. On the other hand, the accuracy (%) between targeted and predicted values for occupancy was 94% for the case where the demand increased by 25%. When the demand increased by 50%, the model still achieved the same accuracy of 94%. The accuracy continues with high values of 95% when future traffic demand is increased by 75% and 100%.

**Table 6:** Summary of results for all scenarios for count, speed and occupancy.

Prediction Horizon	Count (vehicles)			
	25% demand increase	50% demand increase	75% demand increase	100% demand increase
	Prediction Accuracy (%)			
5 Mins	96.31%	97.06%	97.50%	97.76%
10 Mins	94.65%	95.75%	96.43%	96.80%
15 Mins	93.46%	94.62%	95.25%	95.70%
30 Mins	92.20%	93.55%	94.28%	94.79%
45 Mins	90.71%	92.29%	92.85%	93.41%
60 Mins	90.05%	91.46%	92.02%	93.00%

Prediction Horizon	Speed (Km/h)			
	25% demand increase	50% demand increase	75% demand increase	100% demand increase
	Prediction Accuracy (%)			
5 Mins	98.89%	98.80%	98.77%	98.72%
10 Mins	98.80%	98.56%	98.33%	98.29%
15 Mins	98.59%	98.61%	98.61%	98.59%
30 Mins	98.41%	98.31%	98.28%	98.25%
45 Mins	98.02%	98.16%	98.10%	98.04%
60 Mins	94.39%	97.85%	97.70%	97.62%

Prediction Horizon	Occupancy (%)			
	25% demand increase	50% demand increase	75% demand increase	100% demand increase
	Prediction Accuracy (%)			
5 Mins	98.81%	98.86%	98.88%	98.90%
10 Mins	95.33%	95.81%	96.19%	96.23%
15 Mins	93.80%	94.29%	94.68%	94.78%
30 Mins	92.05%	92.58%	92.86%	93.09%
45 Mins	89.06%	90.40%	90.79%	91.37%
60 Mins	88.95%	82.24%	81.82%	81.52%

## Summary of results

This paper developed and successfully calibrated a traffic simulation model using field traffic observations collected from Eastern Freeway in Melbourne, Australia. Simulation results showed that te models replicated field data conditions reasonably well based on GEH and RRMSE criteria. The model was then used to generate large amount of data to develop the prediction models. The results showed BiLSTM achieved high prediction results above 92% up to 60 min into the future for volume count data. For speed, prediction results showed that BiLSTM outperformed other models with an accuracy above 96% up to 60 min into the future. Similarly, occupancy prediction results showed that BiLSTM achieved high prediction results above 92% for up to 60 min into the future. Bi-directional methodology helps extract time-aware traffic information from forward and backward directions. Thus, it helps the traffic prediction model to obtain a better accuracy and our experiments have proved its robustness and efficiency. Melbourne’s travel demand is expected to increase in the future. Hence, it is important that the developed model is able to cope with the future traffic demands. Therefore, the authors took advantage of the calibrated simulation models to evaluate the impacts of traffic growth scenarios on road network performance. For multiple demand increase scenarios, BiLSTM model was used (without re-training) for short-term forecasts up to 60 min into the future. The results showed that BiLSTM is capable of accurate predictions even for future traffic demands that are up to 100% more than baseline year travel demands. The testing of the model without retraining can provide road authorities with confidence that they can apply existing models for future demand changes even if they have not embarked on comprehensive historical data collection efforts. Also, it can assist with reducing the cost of algorithms deployment avoiding the need to pre-process new data and calibrate and validate new models which is a time-consuming undertaking that requires substantial resources and experienced and well-trained AI staff and specialists.

## Conclusions and future research directions

In this paper, Bidirectional LSTM networks were developed to predict traffic counts, speed and occupancy for forecasting horizons up to 60 min into the future. The BiLSTM model was evaluated based on simulated data from a calibrated traffic simulation model of the Eastern Freeway in Melbourne/ Australia. The freeway model was calibrated using field data collected from 55 detectors located along the freeway mainline between July 1, 2016 and August 31, 2016. A comprehensive and rigorous procedure was adopted to match field data with simulated data generated from the software. The results showed that the model was a good fit and was well calibrated on all detector locations across the freeway with GEH < 5 and RMS value of 1.9. Then, the simulated data from the calibrated model were used to predict future speed, counts and occupancy for up to 60 min into the future using BiLSTM. Similarly, a rigorous procedure was adopted to evaluate the suitability of different architectures and modelling parameters. The results showed a superior performance for the Bidirectional compared to Unidirectional LSTM, RNN, Elman and Deep BP models with accuracies above 93% up to 60 min into the future.

This study also evaluated BiLSTM performance on future traffic scenarios when the traffic demand increased by 25%, 50%, 75% and 100%. The results showed that BiLSTM is capable of prediction even if traffic demand increases by up to 100% in the future. For count, speed and occupancy, prediction accuracies were above 92% for all scenarios for a prediction horizon up to 60 min into the future. The results demonstrate the effectiveness of deep learning predictive tools when tested on future traffic pattern changes.

This paper has several shortcomings: the focus of this paper was on the evaluation of traffic state prediction models on freeways only and it doesn’t consider arterial roads. Also, it does not consider other influencing factors such as weather to further refine the prediction models. The study also focused on a congested periods during weekdays only namely, from Monday to Friday. Weekend traffic, which is increasingly becoming an issue in cities like Melbourne due to families using their private vehicles for leisure activities and less reliance on public transport leading to new patterns of congestion over weekends was not included in the prediction analysis. Future research directions include testing the resilience of these developed models on more field data collected from arterial roads and freeways in Australia and overseas. Also, develop and test more architectures to provide a further improved accuracies for a short-term prediction horizon. In addition, investigate the weather impact on the prediction accuracies such as rainfall intensities as a multisource input data.

## Data Availability

To ensure transparency of findings and allow other researchers to audit and reproduce the results reported in this study, the full list of articles considered in literature review can be found on this link: https://drive.google.com/file/d/1DEEZKEW-SsDjCTVELMt2ZJEIHeA2dygn/view?usp=sharing.
